# Staphylococcal protein Ecb impairs complement receptor-1 mediated recognition of opsonized bacteria

**DOI:** 10.1371/journal.pone.0172675

**Published:** 2017-03-08

**Authors:** Hanne Amdahl, Karita Haapasalo, Lydia Tan, Taru Meri, Pentti I. Kuusela, Jos A. van Strijp, Suzan Rooijakkers, T. Sakari Jokiranta

**Affiliations:** 1 Department of Bacteriology and Immunology, Haartman Institute, University of Helsinki, Helsinki, Finland; 2 Research Programs Unit, Immunobiology, University of Helsinki, Helsinki, Finland; 3 Medical Microbiology, University Medical Center Utrecht, Utrecht, The Netherlands; 4 Institute of Biotechnology, University of Helsinki, Helsinki, Finland; 5 Division of Clinical Microbiology, Helsinki University Central Hospital Laboratory, Helsinki, Finland; Pusan National University, REPUBLIC OF KOREA

## Abstract

*Staphyloccus aureus* is a major human pathogen leading frequently to sepsis and soft tissue infections with abscesses. Multiple virulence factors including several immune modulating molecules contribute to its survival in the host. When *S*. *aureus* invades the human body, one of the first line defenses is the complement system, which opsonizes the bacteria with C3b and attract neutrophils by release of chemotactic peptides. Neutrophils express Complement receptor-1 [CR1, CD35) that interacts with the C3b-opsonized particles and thereby plays an important role in pathogen recognition by phagocytic cells. In this study we observed that a fraction of *S*. *aureus* culture supernatant prevented binding of C3b to neutrophils. This fraction consisted of *S*. *aureus* leukocidins and Efb. The C-terminus of Efb is known to bind C3b and shares significant sequence homology to the extracellular complement binding protein [Ecb). Here we show that *S*. *aureus* Ecb displays various mechanisms to block bacterial recognition by neutrophils. The presence of Ecb blocked direct interaction between soluble CR1 and C3b and reduced the cofactor activity of CR1 in proteolytic inactivation of C3b. Furthermore, Ecb could dose-dependently prevent recognition of C3b by cell-bound CR1 that lead to impaired phagocytosis of NHS-opsonized *S*. *aureus*. Phagocytosis was furthermore reduced in the presence of soluble CR1 [sCR1). These data indicate that the staphylococcal protein Ecb prevents recognition of C3b opsonized bacteria by neutrophil CR1 leading to impaired killing by phagocytosis and thereby contribute to immune evasion of *S*. *aureus*.

## Introduction

The human pathogen *Staphylococcus aureus* causes frequently both mild superficial infections such as folliculitis, furunculosis, and impetigo, and more severe invasive infections such as osteomyelitis, endocarditis, and sepsis. In addition, it is a clinical challenge that methicillin resistant *S*. *aureus* strains (MRSA) spread easily leading to healthcare associated infections. *S*. *aureus* is able to colonize distinct microenvironments on skin and mucous membranes and survives inside the human body by expressing several virulence associated proteins. Many of these target the key molecules needed for efficient immune defense, especially the complement system and neutrophils that form the first line of innate immunity against bacteria [1].

The complement system is activated in plasma and other body fluids as a cascade initiated by any of the three pathways, the classical, lectin, or alternative pathway [2]. All pathways converge in proteolytic activation of the central molecule C3, which results in covalent deposition of the C3b fragment onto the target surface. The deposited C3b molecules form new C3-cleaving convertases (C3bBb complexes), which in the absence of efficient down regulation, lead to amplification of the activation and dense and rapid opsonization with C3b [3]. On self cells the amplification is blocked by concerted action of plasma regulator factor H (FH) and cell surface regulators such as complement receptor-1 (CR1, CD35). These regulators act as cofactors for factor I in proteolytic inactivation of C3b to iC3b [4].

In addition to opsonize the target with C3b, further activation of complement leads to initiation of the terminal pathway with release of the powerful anaphylatoxin C5a and formation of membrane attack complexes that oligomerize on plasma membranes and are therefore lytic for Gram-negative but not Gram-positive bacteria [5]. In defense against Gram-positive bacteria C3b-opsonization together with attraction and activation of neutrophils via C5a-receptor (C5aR1, CD88) are important or even essential [6, 7]. Neutrophils recognize the targets to be phagocytosed using receptors for Fc-part of immunoglobulins, receptors recognizing common bacterial structures, and receptors for C3b (CR1, CD35) [8] or iC3b (CR3, CD11b/CD18) [9]. In addition to act as a C3b receptor on phagocytes, CR1 is a complement regulator that acts as a cofactor for factor I in inactivation of C3b. This elongated single chain membrane protein is also found on red cells and it participates in clearance of immune complexes by transporting those on red cells to be eliminated in spleen or liver [10, 11].

*S*. *aureus* is known to secrete several proteins involved in evasion of opsonization and phagocytosis. There are three families of proteins that bind to and impair function of C3b through different mechanisms. The Efb-family includes Efb (*e*xtracellular *f*ibrinogen *b*inding protein) and Ecb (*e*xtracellular *c*omplement *b*inding protein). Both the proteins bind to the C3d part of C3b thereby inhibit formation of new C3 convertases [12–14] and potentiate regulatory function of host FH [15]. The amino-terminus of Efb binds to fibrinogen and impairs platelet functions [16, 17]. Unlike Efb, Ecb is found in all *S*. *aureus* isolates sequenced to date, highlighting its importance in bacterial virulence [18]. Ecb lacks the fibrinogen binding domain but it associates with both FH and C3b to enhance bacterial virulence [15]. Members of the SCIN-family (*s*taphylococcal *c*omplement *in*hibitor), SCIN, SCIN-B and SCIN-C, stabilize and block the activity of the C3-convertase [19–21]. Moreover, SCIN causes dimerization of C3-convertases and it has been shown that FH binding of CR1 and the *c*omplement *r*eceptor of the *Ig* superfamily (CRIg) to such dimeric convertases is impared [19]. The only protein in the third family, Sbi (*S*taphylococcus aureus *b*inder of *I*gG), blocks binding of Fcγ-receptors to IgG and forms tripartite complexes with C3b and FH resulting in down-regulation of complement activation [22, 23]. Recently, it was reported that Efb or Sbi bound to C3b or C3, respectively, together with plasmin enhanced the plasmin mediated cleavage of C3- or C3b-molecule within the complex [24].

Recognition of target-bound C3b deposits by neutrophils is central in opsonophagocytosis. Despite of the central role of opsonophagocytosis in innate immunity there are not, to our knowledge, any reports on microbial molecules that specifically inhibit recognition of monomeric C3b by phagocyte CR1 or even soluble CR1 molecule. Since particularly *S*. *aureus* causes frequently infections where neutrophils are obviously unable to efficiently limit the disease our hypothesis was that it would be the top candidate in having such an immune evasion mechanism. Therefore, we started by studying inhibition of C3b binding to neutrophils by fractions of staphylococcal culture supernatant followed by identification of the molecules that mediate this inhibition. Inhibition of C3b-CR1 interaction for impaired phagocytosis was verified as an immune evasion mechanism using purified proteins in phagocytosis assays highlighting the importance of this new mechanism.

## Materials and methods

### Proteins, sera, and antibodies

The recombinant proteins Efb, and Ecb, the leukocidin components Luk S37, and Luk S45, were expressed and purified as previously described [13, 25]. The FH fragments FH1-4, FH5-7, and FH19-20 were expressed in *Pichia pastoris* [26–28] and FH and C3 were purified from plasma as described previously [15]. C3b was prepared from C3 using trypsin [29]. Soluble CR1 (sCR1) was obtained from CelldexTherapeutics (product code CDX-1135; Needham, MA), and human and bovine serum albumin (HSA and BSA, respectively) were purchased from Sigma-Aldrich (St. Louis, MO). Normal human serum (NHS) was obtained by pooling serum from at least five healthy consented laboratory workers and stored at -70°C until used (Ethical Committee decision 406/13/03/00/2015, Hospital district of Helsinki and Uusimaa). Labeling of sCR1 and C3b was performed with ^125^I (Perkin Elmer, Boston, MA) using the Pierce Iodination Reagent (Thermo Scientific, Rockford, IL) resulting in specific activity of 4.6–8.0 x 10^6^ cpm/μg for sCR1 and 6.0–6.8 x 10^6^ cpm/μg for C3b. The antibodies used were rabbit anti-human C3c (Dako, Denmark), Alexa Fluor® 488-labeled goat anti-rabbit (Invitrogen, Eugene, OR), and FITC-labeled goat anti-human C3 (Protos Immunoresearch, Burlingame, CA).

### Bacterial strains

The strains of *S*. *aureus* used were the Newman strain (from Tim Foster, Dublin) and a strain isolated from a blood culture of a septic patient with the permission of the ethical review board of the Hospital District of Helsinki and Uusimaa, Finland (448/13/03/00/09). A written consent was obtained from healthy individuals. The bacteria were cultured on standard blood agar plates at 37°C under 5% CO_2_ atmosphere.

### Fractionation and identification of proteins from staphylococcal supernatant

*S*. *aureus* strain Newman was cultured overnight in IMDM (Gibco®, Carlsbad, CA) at 37°C and pelleted at 3000 rpm for 15 min. The supernatant was filtered (0.2 μm) and pH adjusted to 7.0 before injected into a HiTrap XL SP Column (GE Healthcare Biosciences; Uppsala, Sweden) that had been washed with buffer (20 mM Na_2_HPO_4_·NaH_2_PO_4_, 140 mM NaCl; pH 7.0). The bound proteins were eluted (20 mM Na_2_HPO_4_·NaH_2_PO_4_, 1 M NaCl; pH 7.0) and separated by size exclusion chromatography (Superdex 75; Pharmacia Biotech, Piscataway, NJ). The molecular weights of the eluted proteins were roughly estimated by injecting a protein marker consisting of con-albumin (75 kDa), carbonic anhydrase (29 kDa), ribonuclease (13.7 kDa), and aprotinin (6.5 kDa). Fractions (500 μl) were collected and their capacity to inhibit binding of neutrophils to C3b was tested. After subjecting the inhibitory fractions to SDS-PAGE, the two most prominent bands were cut out from the gel and analyzed with MALDI-TOF mass spectrometer. The proteins were identified using the Mascot database (Matrix Science).

### Isolation of neutrophils

Neutrophils were isolated as previously described [30] with minor modifications, as follows. Blood was drawn to tubes containing hirudin (Roche Diagnostics, Mannheim, Germany) from healthy human volunteers after informed written and signed consent (Ethical Committee decision 406/13/03/00/2015, Hospital district of Helsinki and Uusimaa). The blood samples were diluted 1:1 with PBS and centrifuged through a gradient (Histopaque® 1.119 and 1.077; Sigma-Aldrich, Steinheim, Germany) at 320 x *g* for 20 min at 22°C. The neutrophil layer was collected, washed once with RPMI 1640 (Gibco®) containing 0.05% HSA (RPMI-HSA), and the remaining red blood cells were lysed with ice-cold water. The isotonic conditions were reestablished with PBS and the cells were washed before diluted with RPMI-HSA.

### C3b binding assays

Two setups were used to analyze the effect of bacterial proteins on binding of C3b to neutrophils. First, to analyze if molecules from the supernatant bound to neutrophils and inhibited C3b-binding, neutrophils (1 x 10^7^ cells/ml) were incubated at 37°C for 30 min followed by incubation for 5 min at 4°C. Undiluted or diluted fractions of *S*. *aureus* supernatant or various concentrations of recombinant proteins of Efb, Luk S37 or Luk S45 (0.01–30 μg/ml) were added to the cells and incubated at 4°C for 30 min. The cells were washed with RPMI-HSA and collected by centrifugation at 1200 rpm for 5 min at 4°C before incubating those with C3b (15 μg/ml; 60 min, 4°C). After washing, the cells were incubated with FITC-labeled goat anti-human C3 (10 μg/ml; 60 min, 4°C) and analyzed by flow cytometer and BD CellQuest Pro software.

Next, we analyzed whether the bacterial protein Ecb could inhibit recognition of C3b by neutrophils in a similar manner as Efb. First, C3b (100 μg/ml) was mixed with Ecb (0.1 or 1 μM) and incubated for 15 min at 22°C before adding 5 x 10^5^ neutrophils to achieve the total volume of 100 μl. After incubation at 4°C for 60 min the cells were washed with RPMI-HSA and incubated with rabbit anti-human C3c (diluted 1:50 with RPMI-HSA) for 20 min at 4°C. The cells were washed and incubated with Alexa Fluor® -labeled goat anti-rabbit antiserum (1:100 in RPMI-HSA) for 20 min at 4°C and the bound antibody was detected by flow cytometry. Forward and side scatters were used to define the neutrophil population and 2000 events were counted. The mean fluorescence intensity was calculated using the Summit software (version 4.3, Beckman Coulter).

### Radioligand assays

The radioligand assays were performed as previously described [15, 31]. In short, 5 μg/ml C3b was coated onto the BreakApart microtiter plate (NUNC, Thermo Scientific, Roskilde, Denmark). The wells were washed three times, and blocked with 0.5% BSA in PBS for 60 min at 22°C. The radiolabeled sCR1 (30,000 cpm) was mixed with various concentrations (0–0.33 μM) of the non-labeled proteins Ecb, FH, FH1-4, FH5-7, FH19-20, or BSA in ½ PBS containing 0.1% BSA in wells of a non-adherent microtiter plate (Greiner Bio-One, Frickenhausen, Germany). An aliquot of the mixture (50 μl) was transferred to the coated wells and incubated for 60 min at 37°C. After washing, the bound radioactivity was measured from separated wells with a gamma counter. The inhibition was calculated as a percentage of bound radioactivity in the presence of the unlabeled protein divided by the radioactivity bound in the absence of it.

### Cofactor assay

To measure the cofactor activity of sCR1 in cleavage of C3b, ^125^I-C3b (100,000 cpm/assay) in PBS was mixed with factor I (125 nM; MerckMillipore, Darmstadt, Germany), and sCR1 (180 nM) in the presence or absence of Ecb (0–2.0 μM). Mixtures (50 μl) were incubated for 15 min at 37°C, and after reducing the samples with β-mercaptoethanol (5 min, 94°C) aliquots were loaded onto a 10% SDS-PAGE gel. The gel was autoradiographed and cofactor activity was evaluated as the intensity of the C3b α’-chain analyzed with GelEval software (FrogDance Software, Dundee, UK).

### Binding of C3b to RBC

Whole blood anticoagulated with EDTA was centrifuged (500 x *g*) for 15 min at 4°C and the plasma, buffy coat, and the uppermost RBC layer were removed. The remaining cells were washed three times with PBS containing 0.5% BSA. In the assay approx. 1 x 10^6^ red cells in 50 μl were incubated with C3b (50 μg/ml) together with Ecb (0–0.1 μM) and incubated at 4°C for 30 min. The cells were washed with PBS and incubated with rabbit anti-human C3c antibody (dilution 1:50) at 4°C for 30 min, followed by washes and incubation with an Alexa Fluor® 488-labeled goat anti-rabbit (dilution 1:100) at 4°C for 30 min. The bound antibody was detected by counting 20,000 events using flow cytometry (CyAN^TM^ ADP).

### Neutrophil binding and phagocytosis assays

Bacteria grown for 17 h in Todd Hewitt broth were washed and labeled with fluorescein coupled to *N*-hydroxysuccinimide-ester (HS-fluorescein, Thermo Scientific; for neutrophil attachment) or pHrhodo^TM^ Green STP ester (Molecular probes, Eugene, OR; for phagocytosis analysis) according to the manufacturer’s protocols. HS-fluorescein was used to measure both binding and phagocytosis of the bacteria whereas pHrhodo label could discriminate phagocytosed bacteria from adherent extracellular bacteria. The labeled bacteria were opsonized by incubation with 20% normal human serum (NHS) for 15 min whereafter the bacteria were washed three times with PBS before the assay. To study whether Ecb could inhibit binding and phagocytosis of neutrophils to *S*. *aureus* the HS-fluorescein labeled and preopsonized bacteria (approx. 5 x 10^3^) were incubated with or without Ecb (0.09 or 0.9 μM) in the presence of neutrophils (approx. 3 x 10^4^) in RPMI-HSA (x μl) for 60 min at 37°C. The pHrhodo labeled bacteria (1 x 10^6^) were incubated with or without Ecb (1 μM or 10 μM) for 10 min at 22°C before incubating with the neutrophils (1 x 10^4^) at 37°C for 60 min on a shaker. To study the effect of sCR1 in Ecb mediated inhibition of phagocytosis the bacteria and neutrophils were incubated with 0.3 μM Ecb and increasing concentrations of sCR1. The concentration of Ecb was chosen according to previous assay to see the effect of sCR1 in this analysis. Incubation without toxin or sCR1 was used as positive control for *S*. *aureus* phagocytosis, and with sCR1 to control that sCR1 alone does not inhibit phagocytosis. The reactions were stopped by adding ice cold RPMI-HSA, and neutrophils were collected by centrifugation (400 x *g*, 10 min). After washing, the cells were fixed with 1% paraformaldehyde for 10 min, and analyzed by flow cytometry.

To analyze the effect of Ecb on neutrophil binding and phagocytosis in whole blood, the HS-fluorescein labeled bacteria (5 x 10^3^ bact) were incubated for 60 min at 37°C with 450 μl of hirudin- or EDTA-anticoagulated blood in the presence of 1.6 μM of the bacterial proteins using an orbital shaker. Hirudin was used since it does not have any effect on complement activation or regulation [32, 33]. The reaction was stopped by centrifugation at 4°C (400 x *g*, 10 min), and red blood cells were lysed with ice-cold aqua, and isotonic conditions were restored with PBS. Following washing and fixing the cells, fluorescence was analyzed by flow cytometry.

### Statistical analyses

The statistical analyses were performed using GraphPad Prism software version 6.0 (GraphPad Software, San Diego, CA) or IBM SPSS statistics and the mean values and SDs of three assays performed with triplicates are presented unless otherwise stated. The radioligand binding curves were fitted using non-linear regression model of the software. Comparison of the mean values was done using an unpaired two-tailed t-test or One-way ANOVA with post-hoc Bonferroni multiple comparison test.

## Results

### Fractions of *S*. *aureus* culture supernatant inhibit binding of C3b to neutrophils

**Fig 1:**
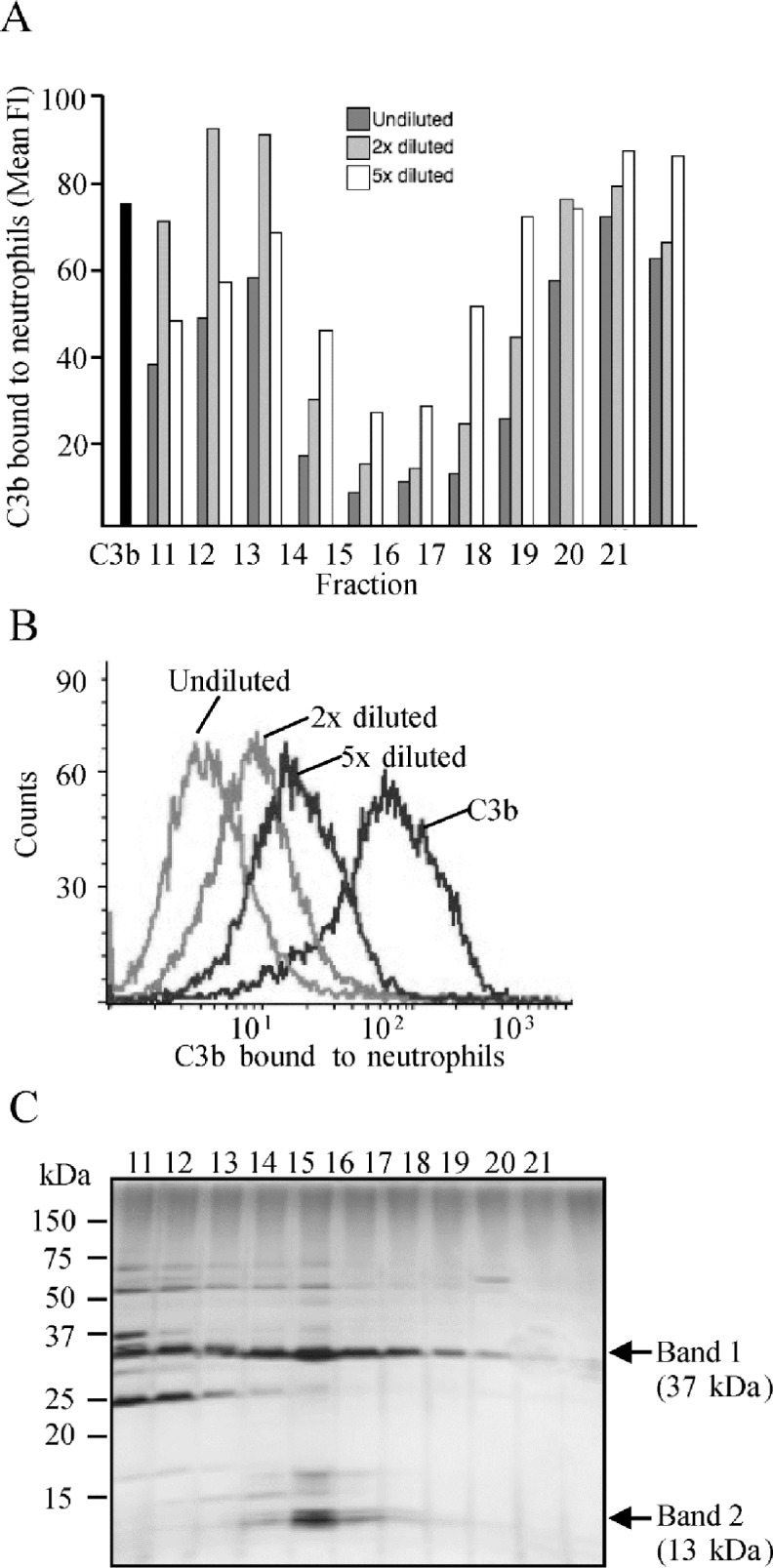
Effect of secreted molecules from *S*. *aureus* on binding of C3b to neutrophils. Fractionated *S*. *aureus* culture supernatant was incubated with C3b and neutrophils and cell-bound C3b was detected with a FITC-conjugated anti-C3 antibody using flow cytometry. *A*, Separated fractions of undiluted, or two or five times diluted culture supernatant were compared. The black bar represents binding of C3b to neutrophils in the absence of culture supernatant. Fl, fluorescence intensity. *B*, Histograms showing the effect of dilutions of the fraction 15 on binding of C3b to neutrophils. *C*, Silver stained SDS-PAGE gel of the fractions 11–21 with molecular weight marker indicated on left and major bands in fractions 15–16 indicated on right.

Neutrophils recognize C3b-opsonized targets with complement receptors. We investigated whether *S*. *aureus* evades opsonophagocytosis by secreting molecules that interfere with this recognition. First, culture supernatant of *S*. *aureus* was fractionated and the effect of each fraction on binding of C3b to neutrophils was tested. While most fractions had no effect the fractions 14–18 inhibited the binding dose-dependently, the strongest inhibitors being fractions 15 and 16 (Fig 1A and 1B). Subsequently molecules in the fractions 11–21 were separated using SDS-polyacrylamide gel electrophoresis and based on silver staining of the gel the fractions 15–16 contained three to five distinct bands at approximately 45, 37, 18, 16, and 13 kDa (Fig 1C). Mass spectrometry of digested peptides from the two most prominent proteins showed best coverage to the S components of the leukocidins LukE (45 kDa, Luk S45) and LukS (37 kDa, Luk S37), and the C-terminus of Efb (16 kDa) (S1 Fig).

### Identification of staphylococcal proteins that impair binding of C3b to neutrophils

**Fig 2:**
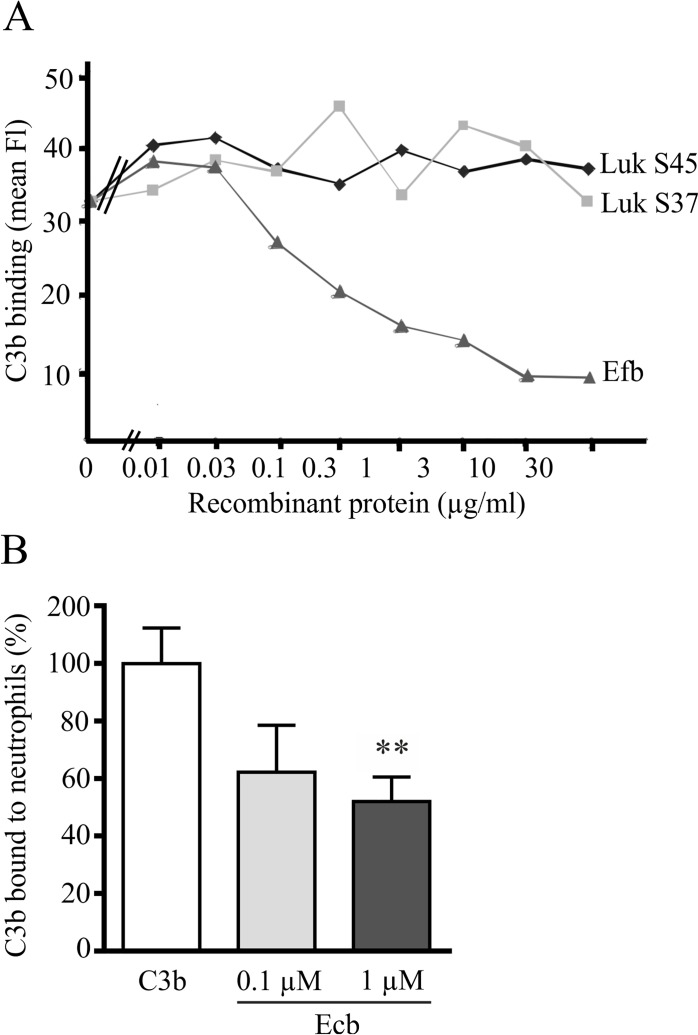
Effect of Efb, Ecb, Luk S37, and Luk S45 on binding of C3b to neutrophils. *A*, Neutrophils incubated with different concentrations of Efb, Luk S37, or Luk S45, were washed before incubation with C3b. The bound C3b was detected with a FITC-conjugated anti-C3 antibody and analyzed by flow cytometry. *B*, Binding of C3b to neutrophils in the presence of two concentrations of Ecb. The bound C3b was detected with anti-C3c and Alexa® Fluor 488 conjugate using flow cytometry. The assay in panel *B* was performed twice in triplicate and after setting binding of C3b in the absence of Ecb to 100%, the shown SD values were calculated. One-way ANOVA with Bonferroni multiple comparison was used to determine the statistical significancies (** *p*<0.01).

Next we tested whether recombinant form of one or more of the three candidate proteins Luk S37, Luk S45, or Efb could impair binding of C3b to neutrophils. A clear inhibition was observed with Efb while no inhibition was observed with the Luk S37 or Luk S45 proteins (Fig 2A). Efb is known to block both complement activation and neutrophil adhesion to fibrinogen and its function in inhibition of platelet aggregation have been very well described [34]. The C-terminus of Efb is highly homologous to Ecb and it binds C3b and blocks complement in a similar fashion as Ecb. [12–14]. However, only Ecb is known to be expressed in all currently sequenced *S*. *aureus* genomes that have been tested for Ecb, and biochemical studies suggests that variants of the same gene may have unique functions and perform differently to enhance bacterial virulence. Therefore, we next focused on studying how Ecb function in *S*. *aureus* innate immune evasion. First we tested whether Ecb similarly to Efb could interfere with C3b binding to neutrophils. Ecb clearly impaired the interaction between C3b and neutrophils in a dose dependent manner so that already at a concentration of 0.1 μM of Ecb the neutrophils bound significantly less C3b than in the absence of the staphylococcal proteins (*p*<0.01) (Fig 2B).

### Ecb inhibits binding of soluble CR1 to C3b

**Fig 3:**
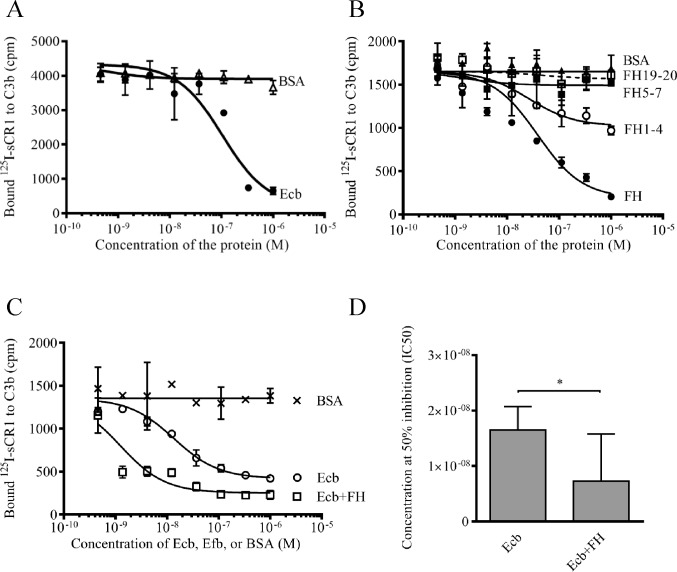
Effect of Ecb and FH on binding of C3b to sCR1. ^125^I-labeled sCR1 was incubated in wells of microtiter plates coated with C3b in the presence of the indicated proteins followed by washings and detection of the bound labeled protein with a gamma counter. *A*, Binding of ^125^I-sCR1 to C3b in the presence of Ecb, or BSA as a control. *B*, Binding of ^125^I-sCR1 to C3b in the presence of FH, or its fragments FH1-4, FH5-7, FH19-20, or BSA. *C*, Binding of ^125^I-sCR1 to C3b in the presence of Ecb alone or together with FH or BSA as a control. *D*, Concentration of the protein needed to inhibit 50% of ^125^I-sCR1 bound to C3b (IC50). The assay in panel *A* was performed three times, assays *B*, *C*, and *D* were performed twice, all with triplicates. Results of representative experiments are shown with mean SD. Differences between means was calculated using One-way ANOVA with Bonferroni multiple comparison (* *p*<0.05).

Since CR1 is found abundantly on neutrophils, we next analyzed whether the Ecb could prevent binding of C3b to neutrophils via inhibition of the interaction between C3b and CR1. In a radioligand assay where binding of labeled sCR1 to solid-phase C3b was measured Ecb inhibited the interaction dose-dependently up to 80% at micromolar concentrations (Fig 3A).

In addition to CR1, also FH binds to C3b [21, 35]. We have recently observed that Ecb and FH enhance binding of each other to C3b on the surface of *S*. *aureus* [15]. Therefore we analyzed whether FH could enhance also the inhibitory effect of Ecb on the interaction between ^125^I-sCR1 and C3b. In the absence of Ecb, FH and its N-terminal fragment FH1-4 inhibited binding of C3b to ^125^I-sCR1 and inhibition was statistically significant for FH (p<0.05 when compared to BSA and other FH fragments) while no inhibition was observed with FH5-7 and FH19-20 (Fig 3B). Ecb could further inhibit ^125^I-sCR1-C3b interaction in the presence of FH (Fig 3C) and statistically significant difference was observed at the highest concentration (p<0.01). IC50-values of Ecb and Ecb together with FH were 1.8x10^-8^ vs. 0.7x10^-8^ (p<0.01) (Fig 3D).

### Cofactor activity

**Fig 4:**
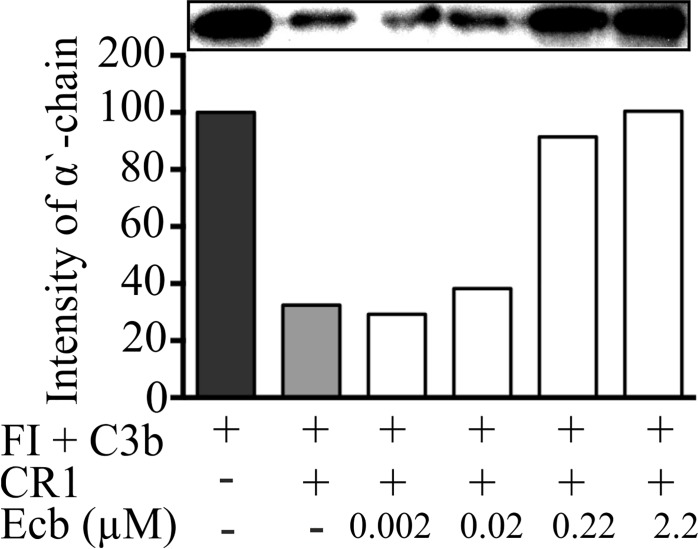
Effect of Ecb on proteolytic cleavage of C3b by sCR1 and factor I. Proteolysis of ^125^I-C3b was analyzed by the intensity of the α’-chain in autoradiography. The intensity in the absence of sCR1 (negative control, black bar), was set to 100% and cleavage in the presence of sCR1 but absence of Ecb was used as a positive control (dark grey bar). The lanes in the upper panel shows the α’-chain in the SDS-page gel. The x-axis shows the presence (+) or absence (-) of the protein and the concentrations (0.002–2.2 μM) of Ecb in the sample.

We have recently shown that Ecb inhibits the cofactor activity of FH in proteolytic inactivation of the α’-chain of C3b by the serine protease factor I. Since also CR1 acts as a cofactor for factor I in C3b inactivation, we next measured whether Ecb has an effect on the cofactor activity of sCR1. We found that Ecb inhibited the cofactor activity of sCR1 already at a 0.22 μM concentration (Fig 4).

### Effect of Ecb on binding of C3b to cell surface-bound CR1

**Fig 5:**
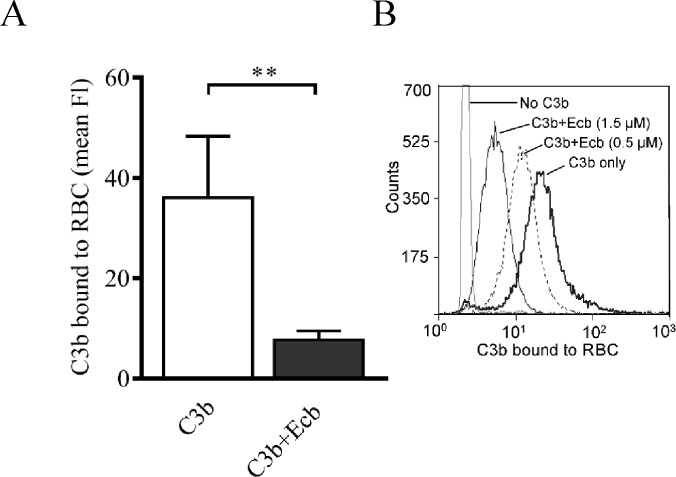
Effect of Ecb on binding of C3b to human red blood cells. *A*, RBC were incubated with C3b with or without Ecb. After washings, C3b bound to RBC was detected with flow cytometry using an antibody recognizing the C3c fragment (FI, fluorescence intensity). The assay was performed three times with duplicates and mean SD values are shown. *B* is a representative histogram of RBC incubated with C3b in the presence of Ecb (0–1.5 and 0–1.8, respectively). Student’s two-tailed *t*-test was used to determine the statistical significances (* *p*<0.05; ** *p*<0.01).

Next we tested whether Ecb could inhibit binding of C3b to cell surface-bound CR1 using red cells devoid of other complement receptors. In the presence of Ecb the binding was impaired (*p*<0.01) (Fig 5A), and the inhibition was dose dependent (Fig 5B). These results indicate that Ecb inhibits binding of C3b to CR1 that is bound to a plasma membrane.

### Ecb together with CR1 efficiently impairs neutrophil phagocytosis

**Fig 6:**
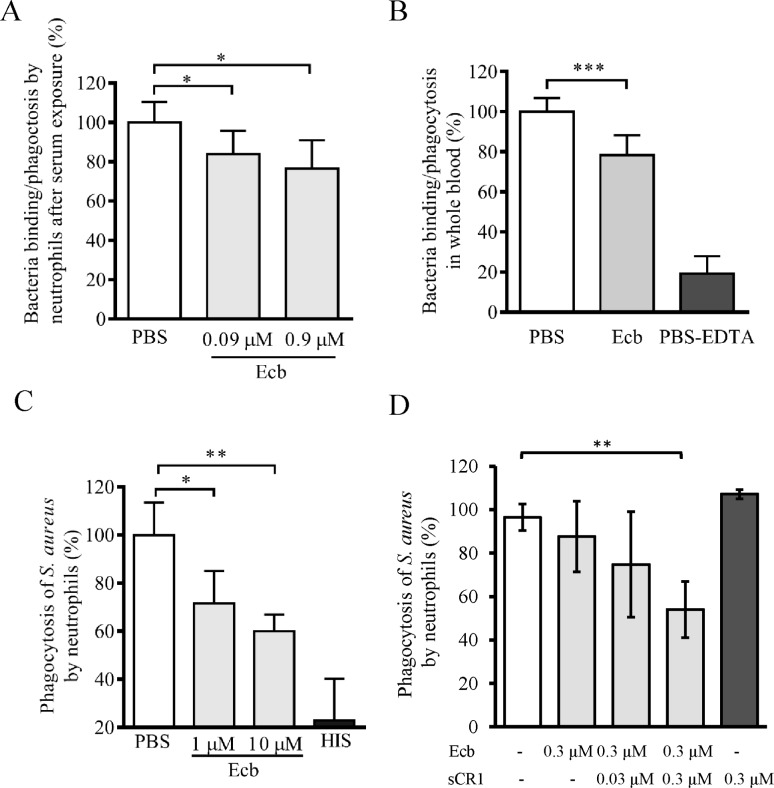
Recognition of opsonized *S*. *aureus* and phagocytosis by neutrophils in the presence of Ecb. *A*, Fluorescence labeled bacteria were opsonized with C3b by exposure to NHS and mixed with Ecb (0–0.9 μM) prior to analysis of neutrophil-bound or phagocytosed bacteria by flow cytometry. *B*, The fluorescent labeled bacteria were exposed to hirudin-anticoagulated blood in the presence of Ecb (1.6 μM) prior to flow cytometry. As a control the bacteria were incubated in blood treated with EDTA that blocks complement activity. *C*, Bacteria labeled with pH rhodo^TM^ were opsonized with C3b by exposure to NHS and incubated with Ecb in the presence of neutrophils. As a control the bacteria were exposed to heat inactivated human serum (HIS, black bar). *D*, The assay in panel *C* was done in the presence of 0.3 μM Ecb and increasing concentrations of sCR1. Sample with only sCR1 was used as negative control and sample without any toxin components as positive control for *S*. *aureus* phagocytosis by neutrophils. The data are from three, four, and two independent experiments, respectively, with mean SD values. Student’s two-tailed *t*-test was used to determine the statistical significancies (* *p*<0.05, ** *p*<0.01, *** *p*<0.001).

To assess the functional consequences of the observation we next tested the effect of Ecb on neutrophil attachment to *S*. *aureus* cells preopsonized with C3b by exposure to NHS. In the presence of Ecb (0.9 μM) binding and phagocytosis of bacteria was significantly decreased to approx. 75% (*p*<0.05) (Fig 6A). Next, we recreated *in vivo* situation during a septic infection by analyzing binding of neutrophils to *S*. *aureus* upon incubation of the bacteria in fresh blood anticoagulated with hirudin and therefore containing full complement activity. In the presence of Ecb (1.6 μM) binding/phagocytosis of the bacteria to neutrophils was reduced significantly (p<0.001, Fig 6B). In the control sample where EDTA was used as the anticoagulant and complement blocking agent the proportion of neutrophils that had bound or phagocytosed the bacteria was only 18% of that seen with hirudin-anticoagulated blood (Fig 6B).

Since labeling of bacteria with HS-fluorescein does not differentiate between neutrophil binding and phagocytosis, we used bacteria labeled with pHrhodo™, a dye that is fluorescent only in acidic conditions such as in the phagolysosome of neutrophils. Ecb inhibited the fluorescence of pHrhodo™ bound to C3b-opsonized bacteria (*p*<0.05 at 1μM, and *p*<0.01 at 10 μM) but not that bound to non-opsonized bacteria (Fig 6C). Because Ecb could reduce binding of C3b to cell bound CR1 and inhibit *S*. *aureus* phagocytosis we next tested whether the molecule could also reduce phagocytosis in the presence of soluble CR1. Inhibition of *S*. *aureus* phagocytosis by Ecb in the absence of active serum was even more efficient when sCR1 and Ecb reached equimolar concentrations (0.3 μM) (Fig 6D) while no inhibition was observed in the presence of only sCR1. This indicates that in the presence of Ecb sCR1 can still bind on C3b in low affinity and inhibit interaction between C3b and cell surface CR1.

## Discussion

Understanding the immune evasion by *Staphylococcus aureus* is critical to identify novel targets for vaccines and thereby to gain control of the infections by this versatile pathogen becoming more and more resistant to current antimicrobials. Phagocytosis is a key mechanism in our defense against pyogenic bacteria such as *S*. *aureus* and in this report we found that this bacterium secretes substances that mediate evasion of opsonophagocytosis. We showed that Ecb could prevent recognition of C3b-deposits by cell surface expressed CR1, and found that the pathogen uses this as a novel mechanism in evasion of phagocytosis.

We have recently described that staphylococcal Ecb and host FH enhance binding of each other to surface deposited C3b [15]. Formation of the tripartite complex, however, did not cause enhanced but inhibited cofactor activity of FH and therefore the C3b deposits were not turned into enzymatically inactive iC3b deposits. This was surprising since C3b opsonization is known to make a particle readily phagocytosed [36, 37] and could lead to propagation of the complement activation resulting in MAC formation and potentially lysis of the target [38, 39]. Since the used blood culture derived *S*. *aureus* strains are clearly pathogenic we hypothesized that C3b on *S*. *aureus* cannot be opsonic but that this pathogen must have a way to prevent recognition of C3b-deposits by phagocytes.

In this report we started by studying whether there are factors in staphylococcal culture supernatant that inhibit binding of C3b to phagocytes. We had two reasons to assume that the possible phagocytosis inhibitors are secreted and not primarily membrane proteins. First, secreted inhibitors could prevent efficiently recognition of C3b even though this opsonic molecule was deposited onto any surface structure, even to those distant from the plasma membrane. Second, by secreting the immune evasion molecules a bacterium could prevent recognition of such molecules on its own surface by antibodies later during the infection. The bacterium with most identified secreted immune evasion molecules is *S*. *aureus* (reviewed in [1, 40] and this could indicate that secreted molecules are particularly useful for this pathogen, perhaps explaining the often chronic or long lasting infections by *S*. *aureus*.

Even in a nonimmune individuals the alternative pathway of complement leads to fast deposition of C3b-molecules onto the surface of foreign targets followed by opsonophagocytosis by neutrophils and macrophages. Since the interplay between complement and neutrophils is a rapid and powerful defense, several bacteria have developed ways to inhibit this. One way for a bacterium to protect itself is to acquire the complement regulator FH onto its surface. Binding of FH to all the more than ten FH-acquiring microbes has led to enhanced degradation of C3b to iC3b (e.g. [41–45]. This is generally considered to be only beneficial since cleavage of C3b to iC3b prevents propagation of the activation cascade. But upon cleavage of C3b to iC3b a ligand is formed for complement receptor 3, i.e. CR3 (CD11b/CD18), found in high numbers on macrophages and neutrophils, and CR4 (CD11c/CD18) found on macrophages, neutrophils, and dendritic cells. Therefore cleavage of C3b to iC3b could also be hazardous for the microbe [36].

In staphylococcal complement evasion the potential benefit of inhibiting iC3b formation becomes relevant since C3b bound to Ecb or Efb is known to be unable to form the C3- and C5-convertases and to be cleaved to iC3b by factor I. Therefore the only remaining threat that the C3b-deposits create for the staphylococci is that these deposits are powerful opsonins [46]. CR1 on neutrophils binds C3b-opsonized particles and facilitates phagocytosis by stimulating CR3 and CR4 [47]. Expression of CR1 on neutrophils is increased upon stimulation [48], especially during a bacterial infection [49]. The importance of CR1 for phagocytosis *in vivo* is demonstrated in a mouse model where loss of CR1 strongly contributed to survival of *S*. *aureus* in the mouse blood [50].

To our knowledge *S*. *aureus* is the only bacterium that inhibits directly binding of CR1 to C3b. Previously, it was described that dimerization of C3-convertases by SCIN could impair the C3b-CR1 interaction [19]. Since C3 is abundant in plasma (0.7–1.4 mg/ml) and C3b is quickly deposited onto the bacterial surface [37] it is essential for the bacteria to have multiple ways to avoid opsonophagocytosis. SCIN acts as a rescue of the already formed C3-convertases, while Efb and Ecb inhibit C3b also before the convertases are formed. Our results show that Ecb blocks the cofactor activity of soluble CR1 leading to decreased iC3b formation, similar to that we described for FH. But, unlike FH, only CR1 acts as a cofactor for factor I in cleavage of iC3b to C3d and C3c. Because we observed further increase in phagocytosis inhibition when both Ecb and sCR1 were present, even in the absence of active serum, we hypothesized that Ecb inhibits but does not completely block C3b-CR1 interaction. This indicates that the function of Ecb is to reduce phagocytosis by reducing iC3b and C3d formation and thereby recognition by neutrophil receptors. Inhibition of C3d formation could have consequences also for the adaptive immunity since the C3d-deposits on foreign targets are recognized by CR2 on the antibody producing B cells linking the innate and adaptive immunity [51, 52]. Therefore the inhibition of the cofactor activity of CR1 by Ecb could slow down initiation of the adaptive immune response against *S*. *aureus*. In addition to this, it has previously been shown that the C-terminus of Efb binds to the CR2 binding site on C3b thereby directly inhibiting the C3d-CR2 interaction [53].

When staphylococci were incubated with whole blood, Ecb impaired binding of the bacteria to neutrophils (Fig 6B). The involvement of Ecb in evasion of opsonophagocytosis in full blood is in line with a previous study where both Efb knockout and Ecb knockout strains became sensitive to killing in whole blood and the double knockout strain was the most sensitive [54]. When staphylococci were preopsonized with normal human serum (not full blood) Ecb inhibited binding of the bacteria to neutrophils (Fig 6A) even in the absence of active serum.

Taken together, the human pathogen *S*. *aureus* is very well adapted to its host and uses several ways to evade or modulate the immunity by targeting different steps of the complement cascade and phagocytosis. Due to the increasing antibiotic resistance of *S*. *aureus* it is important to be aware of all the immune evasion strategies to choose the most suitable ones for development of novel vaccines and/or antimicrobials. The secreted small proteins Ecb is expressed by all currently investigated clinical isolates highlighting the importance of the molecule in *S*. *aureus* immune evasion. By binding to C3b Ecb do not only inhibit the complement activation but, as shown in this report, also inhibits recognition of C3b by CR1 thereby preventing CR1-mediated phagocytosis of *S*. *aureus*. Prevention of these functions are likely to make the bacteria clearly more susceptible for our defense mechanisms. Therefore, although Ecb is not a membrane protein as traditional vaccine components, we consider this protein as rational and innovative vaccine candidate.

## Supporting information

S1 Fig Sequence results of the two bands cut out from the SDS-PAGE gel after size exclusion chromatography. Band 1 had 65% sequence coverage to the C-terminus of Efb while band 2 gave 39% and 38% sequence coverage to components of the Leukocidins LukS and LukE, respectively.
(PDF)
