# Supplementary material for: Staphylococcal protein Ecb impairs complement receptor-1 mediated recognition of opsonized bacteria
Source: PLoS One. 2017 Mar 8;12(3):e0172675. doi: 10.1371/journal.pone.0172675 (PMC5342210; doi:10.1371/journal.pone.0172675)
Supplement: S1 Fig — Band 1 had 65% sequence coverage to the C-terminus of Efb while band 2 gave 39% and 38% sequence coverage to components of the Leukocidins LukS and LukE, respectively. (PDF) [file pone.0172675.s001.pdf]

Band 1

Protein name: Chain A, Crystal Structure Of Efb-C From *Staphylococcus aureus*
Alphalyse number: ALPHA09150
GI-number: gi|145579437
MW: 7184
pI: 10,23
Mascot score: 179
Sequence coverage: 65%

1 IKKEQK**LIQA QNLVREFEKT HTVSAHR**K**AQ KAVNLVSFEY K**VKK**MVLQER**
51 IDNVLKQGLV R

Band 2

Protein name: leukocidin LukS component [*Staphylococcus aureus* subsp. *aureus*]
Alphalyse number: ALPHA09148
GI-number: gi|13549150
MW: 32563
pI: 9,09
Mascot score: 273
Sequence coverage: 39%

1 ANDTEDIGKG SDIEIIKRTE DKTSNK**WGVT QNIQFDFVK**D KKYNK**DALIL**
51 **KMQGFISSRT TYYNYK**KTNH VKAMR**WPFQY NIGLK**TNDK**Y VSLINYLPK**N
101 KIESTNVSQT LGYNIGGNFQ SAPSLGGNGS FNYSKSISYT QQNYVSEVEQ
151 QNSKSVLWGV KANSFATESG QK**SAFDSDLF VGYKPHSKDP R**DYFVPDSEL
201 PPLVQSGFNP SFIATVSHEK **GSSDTSEFEI TYGR**NMDVTH AIKR**STHYGN**
251 **SYLDGHRVHN AFVNR**NYTVK **YEVNWK**THEI KEKGQN

Band 3

Protein name: leukotoxin, LukE, putative [*Staphylococcus aureus* subsp. *aureus* NCTC 8325]
Alphalyse number: ALPHA09148
GI-number: gi|88195648
MW: 33666
pI: 9,33
Mascot score: 294
Sequence coverage: 38%

1 MSVGLIAPLA SPIQESRANT NIENIGDGAE VIKRTEDVSS KK**WGVTQNVQ**
51 **FDFVK**DKKYN K**DALIVKMQG FINSR**TSFSD VKGSGYELTK R**MIWPFQYNI**
101 **GLTTKDPNVS LINYLPK**NKI ETTDVGQTLG YNIGGNFQSA PSIGGNGSFN
151 YSKTISYTQK SYVSEVDKQN SKSVKWGVKA NEFVTPDGKK SAHDR**YLFVQ**
201 **SPNGPTGSAR** EYFAPDNQLP PLVQSGFNPS FITTLSHEK**G SSDTSEFEIS**
251 **YGRNLDITYA TLFPRTGIYA ERKHNAFVNR** NFVVR**YEVNW K**THEIKVKGH
301 N
